# Medicinal Plant Growth in Heavy Metals Contaminated Soils: Responses to Metal Stress and Induced Risks to Human Health

**DOI:** 10.3390/toxics10090499

**Published:** 2022-08-27

**Authors:** Raluca Maria Hlihor, Mihaela Roșca, Laura Hagiu-Zaleschi, Isabela Maria Simion, Gabriel Mihăiță Daraban, Vasile Stoleru

**Affiliations:** 1 Department of Horticultural Technologies, Faculty of Horticulture, “Ion Ionescu de la Brad” Iasi University of Life Sciences, 3 Mihail Sadoveanu Alley, 700490 Iasi, Romania; 2 Department of Organic, Biochemical and Food Engineering, “Cristofor Simionescu” Faculty of Chemical Engineering and Environmental Protection, “Gheorghe Asachi” Technical University of Iasi, 73 Prof. D. Mangeron Blvd., 700050 Iasi, Romania

**Keywords:** detoxification mechanisms, medicinal plants, reactive oxygen species, plant responses to abiotic stress, toxic metals

## Abstract

Accelerating heavy metal pollution is a hot issue due to a continuous growth in consumerism and increased activities in various global industries. Soil contamination with heavy metals has resulted in their incorporation into the human food web via plant components. Accumulation and amplification of heavy metals in human tissues through the consumption of medicinal plants can have hazardous health outcomes. Therefore, in this critical review we aim to bring together published information on this subject, with a special highlight on the knowledge gaps related to heavy metal stress in medicinal plants, their responses, and human health related risks. In this respect, this review outlines the key contamination sources of heavy metals in plants, as well as the absorption, mobilization and translocation of metal ions in plant compartments, while considering their respective mechanisms of detoxification. In addition, this literature review attempts to highlight how stress and defensive strategies operate in plants, pointing out the main stressors, either biotic or abiotic (e.g., heavy metals), and the role of reactive oxygen species (ROS) in stress answers. Finally, in our research, we further aim to capture the risks caused by heavy metals in medicinal plants to human health through the assessment of both a hazard quotient (HQ) and hazard index (HI).

## 1. Introduction

Medicinal plants have been used since ancient times in different kind of therapies. Using plants as an alternative to conventional medicine is considered by a large part of the population to be safer, less toxic for the human body, more easily available, and more accessible. However, due to the continuous increase of environmental pollution caused by the demands of consumerism, researchers have come up with high concerns on safety and quality issues related to the use of herbal products [1,2]. Inorganic pollutants, such as heavy metals, represent a wide interest category of pollutants for risk studies due to their impacts on the environment and on human health [3,4]. According to the World Health Organization (WHO), heavy metals are some of the most dangerous pollutants; non-degradable, they bioaccumulate in the environment, transfer through the food chain, and induce negative effects on the environment and human health [5]. The metals environmental chemistry strongly influences the effects on human and ecological receptors. In the environment, under the action of abiotic and biotic factors, heavy metals can be transformed from one species to another (e.g., their valence state is changed) and can be converted between inorganic and organic forms. Also, metals are present in environmental compartments in different sizes, from small particles to large masses [6].

Considering these aspects, a possible contamination with heavy metals of plants used in phytotherapy and cosmetology for specific products could have risks to human health, even in low concentrations. For example, heavy metals found in cosmetic products can act locally on skin or can accumulate inside the human body after absorption and could cause systemic toxic effects [7]. There are several ways in which heavy metals can enter the human body, including inhaling dust, ingesting soil, dermal contact, and consuming plants grown on contaminated soil. In the human body, heavy metals have an increased risk of cardiovascular, neurological, and renal diseases [8,9,10].

Works in the available literature highlights the use of different types of plants for medicinal purposes over time around the world. As estimated by the World Health Organization (WHO), approximately 25% of modern medicine has been derived from plants being used in traditional medicine, while there is an estimate of 80% of the world’s population using medicinal plants as the main form of health [11,12]. Traditional herbal remedies are prepared in several ways, some of which are infusions, decoctions, tinctures, or macerations. The increased interest for the benefits of using medicinal plants has led to the expansion of the fields of use, such as phytotherapy, aromatherapy, perfumery, manufacture of products for personal care, gastronomy, cosmetology [13,14], or as bioinsecticides [2,15,16].

The role of medicinal plants is not only limited to traditional medicine. Due to increasing demand of plant-based products in industries such as foods, pharmaceuticals, essential oils, cosmetics, and even ornaments, as well as environmental counterbalances to industrial and agriculture pollution, the economic value of these plants has been improved [17,18]. According to Pruteanu and Muscalu [18], medicinal plant abilities are different and a selection of them have demonstrated the ability: (i) to tolerate elevated levels of heavy metals and accumulate them in very high concentrations; (ii) to remove, contain, inactivate or degrade harmful environmental contaminants, such as: Cd, Ni, Pb, Zn, Cr; (iii) to absorb a high number of elements from soil and water; and (iv) to be compared to solar driven pumps which can extract and concentrate several elements. Practically, it was determined that the chemical composition of any plant depends upon the local geographical conditions, type of soil and its composition.

Herbs are even considered a good choice for phytoremediation, as they are grown primarily for processing and also have the capacity to accumulate and eliminate heavy metals from environment, as well as to reduce risks on human health [19,20]. Angelova [21] used *Lavandula angustifolia* (lavender) in phytoremediation and the results showed that heavy metals accumulated by this species are not transferred in the essential oils. The possibility of further industrial processing makes lavender an economically interesting crop for farmers considering the phytoextraction technology.

In the context of current pollution, the need for research regarding the content of different chemicals such as heavy metals in plants intended for consumption or use is imperative. By self-harvesting medicinal plants in areas at risk of being contaminated with various pollutants (areas with heavy road traffic, heavily industrialized areas, acid rain, etc.), human health can be endangered, to the detriment of the benefits offered by these plants. We propose a systematic review focusing on the challenges posed by the interactions between medicinal plants and heavy metals. Therefore, in the present study, we initially focus on the background and related studies concerning identifying the sources and pathways leading to medicinal plants contamination with heavy metals. Further, phytotherapy and cosmetics, as paths for human health risks caused by contaminated medicinal plants, have been reviewed. We continue with stress factors and the defense mechanisms given by the antioxidant activity of plants and finally, health risks due to heavy metal toxicity have been discussed by the identification of the hazard quotient (HQ) and hazard index (HI) assessed for heavy metals in different medicinal plants.

## 2. Related Studies and Background

The human health risk assessment of plants exposed to heavy metals and used in different purposes represents a challenge for researchers, evaluators, and risk managers, in particular considering the limitations of current methodologies concerning the relationship between terminology and problem formulation, absorption mechanisms, toxicological profile, and human exposure conditions. These situations underline the importance and difficulty of the problem from all points of view. Until now, most studies have aimed to investigate the concentrations of metals in plants that cause toxicity [20,22] without taking into account the safety levels of metals for human intake.

For example, Adedokun et al. [23] studied the potential human intake of heavy metals, including in their work different types of population, such as people with skinny and tiny body types, with high health sensitivity, pregnant women, and people consuming vegetables poisoned with different heavy metals (Pb, Cr, Zn, Cu, Cd, Ni). To assess the risks to the target population, this group of researchers had taken into consideration several indicators such as health risk index (HRI), daily intake of metal (DIM), and the target hazard quotient (THQ). Considering the order Pb > Cu > Cd > Ni > Zn > Cr, the authors concluded that the inhabitants of Lagos consuming leafy vegetables from the market are highly exposed to risks associated with the identified metals. According to the collected data, it is necessary to be more careful when dealing with sensitive population such as pregnant women, people with weak immune systems, and others when heavy metals uptake is higher than for the normal population [23].

Shokri et al. [24] studied the human health risk posed by heavy metals through onion consumption. *Allium cepa* is a widely used vegetable throughout the world and is also used for its medical benefits such as its anti-cancer, antimicrobial, antiviral, antifungal properties. Extracts and essential oils of these plants are effective in treating cardiovascular diseases. Cadmium concentration in onion samples was found to be higher than the standard specification of cadmium content in onion for Iran (50 μg kg^−1^). The lead concentration found in samples was below the maximum allowable limit specified by WHO-FAO (300 µg kg^−1^). The authors found that the quantities of heavy metals identified in onion samples do not pose a carcinogenic risk [24].

Zhang et al. [25] determined the concentration of arsenic and cadmium in the herbal medicine *Panax notoginseng* (PN), which is largely consumed in China. The medicinal plants taken into study had a determined concentration of Cd and As between 0.07 and 1.97 mg/kg and 0.11 and 1.26 mg/kg, respectively. For gastrointestinal bioaccessibilities, the values were between 64.7 ± 3.9% for As and 84.1 ± 10.1% for Cd. The authors estimated that the consumption of PN may lead to carcinogenic risks to 3.53% of consumers. The safety limits for PN with metal content were calculated to be 3.6 mg/kg for Cd and 1.5 mg/kg for As. The safety limits for content in soil was determined to be 5.5 mg/kg for As and 7.3 mg/kg for Cd [25].

Zárate-Quiñones et al. [26] determined the impact to human health of As, Pb, and Cd found in *Eucalyptus globulus* (used as treatment of respiratory diseases) and *Minthostachys mollis* (analgesic, expectorant, anti-inflammatory, antispasmodic, and antiasthmatic) from Peru. The identified values of heavy metal content in the studied plants suggested that there is no health risk. The researchers conclude that it is useful to pay more attention to the risk posed by Cd and Pb to human health due to the consumption of infusions, especially those of *E. globulus* leaves [26].

**Figure 1:**
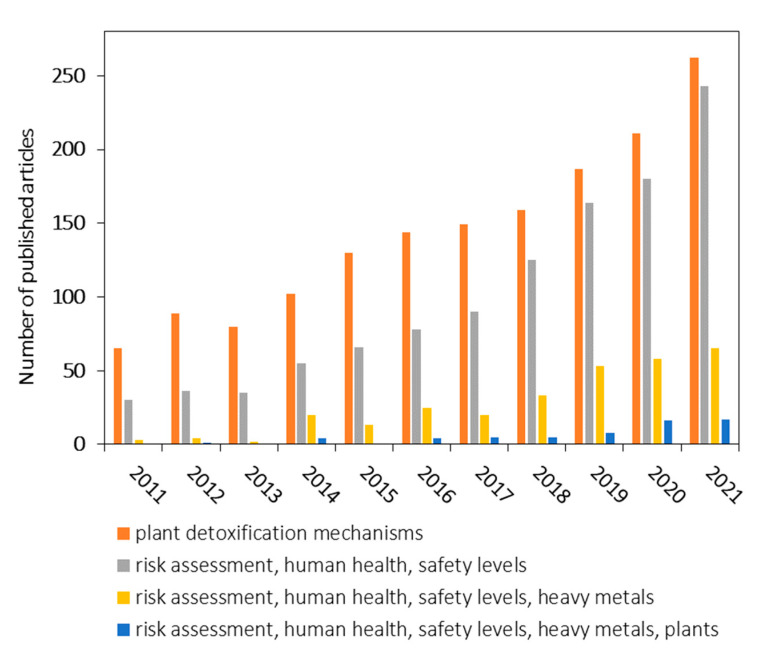
A structured literature review according to data from Web of Science database comprising of the link between plant detoxification mechanisms and the risks posed to human health given the safety levels of heavy metals in plants.

Given these considerations, we performed a detailed search over the years 2011 to 2021 in the Web of Science database, to investigate the scientific literature availability on specific links in the current topic (Figure 1). The research question that was addressed was: “Are there any connections in the scientific literature comprising of plant detoxification mechanisms and the risks posed to human health given the safety levels of heavy metals in plants”?

The assessed keywords leading to possible answers and the related number of articles as estimated in June 2022 were: (1) plant detoxification mechanisms with a total of 1578 articles; (2) risk assessment, human health, safety levels with a total of 1102 articles; (3) risk assessment, human health, safety levels, heavy metals with a total of 296 articles; and (4) risk assessment, human health, safety levels, heavy metals, plants with a total of 60 articles. We also included all of the specified keywords in one search which gave us zero results. The limitations of the current approaches are given by the fact that there are no studies in literature that focus on the link between plant detoxification mechanisms and the risks posed to human health to establish safety levels of heavy metals in plants for human intake. Therefore, it is very important to present a complete picture of the presented information related to the behavior of heavy metals in medicinal plants and the risks to human health to establish potential safety levels.

## 3. Medicinal Plants Contamination with Heavy Metals: Sources and Pathways

Particles loaded with heavy metals resulted from technological processes which are released into the atmosphere can cause environmental pollution at different intensities, depending on the distance from the emission source, the topography of the terrain, and the prevailing winds direction [5]. Most metals and metal compounds occur almost exclusively in the particle phase of the atmosphere due to the fact that they exist in solid phase under normal environmental conditions. Anthropogenic sources of air pollution with heavy metals include the burning of fossil fuels and the metal industry and various industrial activities that use specific metal compounds in specific processes. For example, manganese and nickel can come largely from bark sources [5,27]. It turned out that, in the atmosphere, fine and coarse particles usually behave differently. The fine particles have a longer persistence (e.g., days, weeks) than large particles and tend to be more evenly dispersed over large geographical regions. The relatively low deposition rate of fine particles contributes to their persistence and uniformity throughout the air mass, while coarser particles, larger than 10 µm, tend to fall rapidly out of the air and depending on their size and other factors, their atmospheric lifetimes can be from minutes to hours [28].

In most cases, heavy metals are not subjected to particle phase transformation, and thus their removal from the atmosphere is correlated with the participation rate of the particles in wet and dry processes deposition. In airborne compounds, the heavy metals are generally found with a single predominant oxidation state. For example, the oxidation state of metals such as Be, Cd, and Pb is always 2+. As result of industrialization, currently metal levels may be higher than those naturally existing [29].

In soil, heavy metals occur naturally as a result of pedogenic processes of parent materials alteration and their levels are under 1000 mg/kg [30]. Rapid industrialization and human activities have accelerated the geochemical cycling of metals but have also released significant quantities in all environmental compartments. All of these actions have led to increase of heavy metals levels above defined background values, most in rural and urban soils. Lead (Pb(II)), chromium (Cr(III)), arsenic (As(II)), zinc (Zn(II)), cadmium (Cd(II)), copper (Cu(II)), mercury (Hg(II)), and nickel (Ni(II)) are the most commonly heavy metals ions found in contaminated soils [30,31]. Land applications of inorganic fertilizers, manure, sewage sludge, pesticides, disposal of waste, mine tailings, road traffic, etc. are among the anthropogenic sources of heavy metal soil pollution [30,32]. According to De Vries [33] in the agriculture lands the use of mineral phosphate fertilizers is the main source for cadmium, the animal manure inputs copper and zinc, and via sewage sludge uses, lead, cadmium and zinc are introduced into soil. At the European level, annually the use of inorganic fertilizers inputs maximum 1.21 g/ha Cd, 7.1 g/ha Cu, 15 g/ha Pb and 48.3 g/ha Zn. Via animal manure in the grassland and arable land maximum of 0.86 g/ha Cd, 181.1 g/ha Cu, 17.5 g/ha Pb and 800.1 g/ha Zn are introduced [33]. In 2015, Bai et al. [34] reported that the maximum contents of Cd, Zn, Cr, Cu, Pb and Ni in fertilizers and manure applied to greenhouse soil in Wuwei District, China were of 37.69, 4463.7, 380.88, 1007.84, 127.40 and 28.32 mg/kg, respectively. The results of the study conducted by Defarge et al. [35] showed that in the glyphosate-based herbicides (GBH) were detected in particular As, Cr, Ni, Pb and Co. For example, in 6 GBH the As permitted level in water was exceeded even after recommended dilutions (1.5–15%) for agricultural or garden applications. Disposal of waste is also a source of soil contamination with heavy metals due to leachate migration. According to the study carried out by Kanmani and Gandhimathi [36], in the leachate samples were detected maximum 1.0372 mg/L Cd, 2.6886 mg/L Cu, 1.7962 mg/L Mn and 5.1485 mg/L Pb, respectively. In the soil samples collected from a distance of 200 m of the open dump site boundary the maximum levels of Cu, Cd, Mn and Pb were 39.27 mg/kg, 43.63 mg/kg, 156,14 mg/kg, and 291.29 mg/kg.

The pathway of heavy metals in the environment from source to emission destination makes the soil the main reservoir of heavy metals released into the environment as a result of anthropogenic activities, and therefore it can pose risks and hazards to humans and the ecosystem. Tóth et al. [37] estimated that 6.24% (137,000 km^2^) of European agricultural land are improperly for food production due the contamination with heavy metals. According to Wuana and Okieimen [30] contamination of soil with heavy metals reduces the land use capacity for agricultural production and the food quality through phytotoxicity. Heavy metals contaminated soil is a hazard to humans, since the ions can enter the human body as a result of direct ingestion or dermal contact with the soil, drinking of contaminated groundwater or through the food chain (soil-plant-human or soil-plant-animal-human bioaccumulation). Several studies highlighted that the accumulation of heavy metals in plant organs inhibit the growth and productivity of plants, sometimes it even causes their death [38,39,40]. For example, As, Cd, Pb, Hg, and Zn negatively affected the seed germination of rice, wheat, maize, cluster bean. Chibuike and Obiora [39] also asserted that heavy metals caused chlorosis, a decrease in plant nutrient content, reduced the shoot and root length, damaged the leaf area and dry matter production, and had other negative effects.

**Figure 2:**
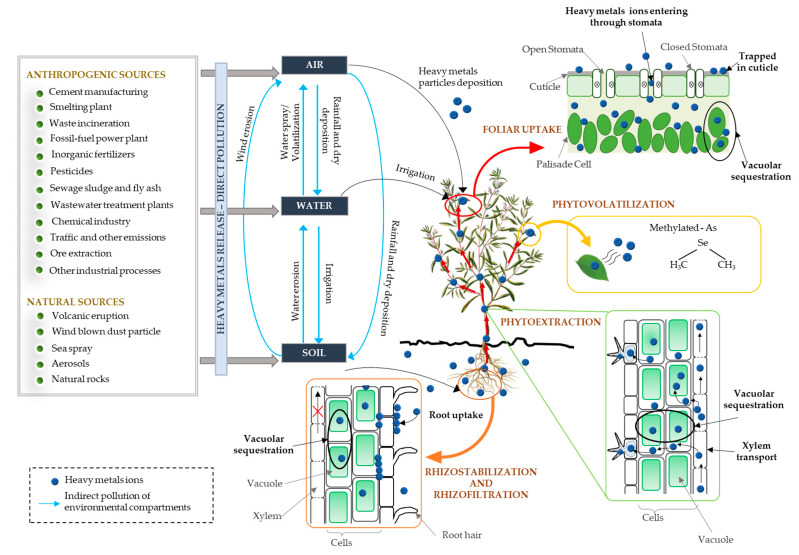
Sources of plants contamination with heavy metals and uptake, mobility and translocation of ions in plant parts.

The variety of natural and anthropogenic sources as well as the exchanges between the environmental components led to the contamination of the all-environmental compartments with different pollutants, including heavy metals [32,41]. Environmental pollution inevitably causes plant contamination and the absorption of metals by plants occurs through soil, water or air (Figure 2), and as they advance through the food chain, the metals tend to concentrate in living organisms and can affect human health.

Accumulation of heavy metals in plants is due the uptake by root and/or leaves, their absorption, translocation, and compartmentation being involved several mechanisms, which are closely related to the pollution sources and the exposure pathway. Metal ions’ distribution in plants parts vary with plant species and could be influenced by the anatomic and biologic peculiarities of the plants [42].

The heavy metals uptake by roots depends of the physico-chemical properties of soils which may influence the metal bioavailability. The pH, soil organic matter, cation-exchange capacity, and soil microbial activity are among the most important parameters. For example, the mobile fractions of heavy metals are higher in soils with acidic pH [43], since at low pH, the heavy metals are usually found as free ionic species or as soluble organometals [44,45]. According to Olaniran et al. [44], in organic soils the heavy metals forms are less mobile and less bioavailable for plants uptake than metals forms present in mineral soils. A part of soil microbiota can improve the uptake and translocation of metals by producing and secreting various organic acids, polymeric compounds, chelators, and hormones which are responsible for soil pH decrease and metal bioavailability enhancement. Other microbial species, by secretion of polymeric compounds, help in the immobilization of metals by decreasing their mobility [46,47].

As response to heavy metals stress, the plant roots release a variety of exudates that can reduce/increase the bioavailability of metals in soil by changing the pH or metal–metabolite complexes formation [48,49]. Therefore, the exudates released by plants either immobilize the heavy metals within the rhizosphere or favor the penetration of metal ions inside the root cells, where the ions can be immobilized in the extracellular space (apoplastic cellular walls) or in vacuoles. This plant mechanism is known as phytostabilization and through this, plants are able to prevent the possible off-site contamination of metal migration through soil dispersion, leaching, water erosion and wind. In other cases, the plants roots absorb and sequester the metals ions from contaminated land surfaces or groundwater, then concentrate and/or form precipitate heavy metals through their root system or other submerged organs, a mechanism known as rhizofiltration [50,51].

The metal ions sequestered inside the root cells vacuoles subsequently may be transported into the stele; through the root symplasm they enter into the xylem stream. Afterwards, the ions are translocated to the shoots via xylem vessels. From shoots through apoplast or symplast, the metals are transported to leaves where are sequestered in extracellular compartments (cell walls) or plant vacuole. Thus, the accumulation of metal ions in cytosol is avoided. By sequestration and compartmentalization in vacuoles, the plant cells are protected against the harmful effects of heavy metals, as they are removed from the cell sensitive areas where cell division and respiration take place. Furthermore, the interactions between metal ions and cellular metabolic processes are reduced and the cell functions damages avoided. Besides vacuoles, other locations where the heavy metals can be sequestrated and their accumulation cause less damage to the plant are leaf petioles, leaf sheathes and trichomes [50,51,52].

Accumulation of heavy metals in the leaves could be also due the foliar uptake as result of the deposition of heavy metals particles from the air on the surface of the leaf and of the contaminated water particles which stayed on the leaves after irrigation [53,54]. According to Shahid et al. [54] the heavy metal may enter inside the plant leaves through stomata, pores present on the leaf cuticle, lenticels, ectodesmata, and aqueous pores. The metals ions adsorbed on plant leaves are mainly retained by trichomes and cuticular waxes, but a part of these can enter inside plant leaf tissues and then transported to root and other organs [51,54].

Sometimes the heavy metals absorbed by roots and transported to the leaves are converted into non-toxic forms and safety released into the atmosphere, as volatile hydride and methyl compounds. This process is called phytovolatilization and is driven by the evapotranspiration of plants. The plants use this mechanism in detoxification of heavy metals such as Se, Hg, and As [50,55].

Deep research showed that the accumulation of heavy metals in medicinal plant tissues varies based on plant species and metal elements. Due to adaptation mechanisms of medicinal plants it results a good survival capacity under a highly polluted environment [17]. For example the results obtained by Angelova [56], indicated that the roots of *Ocimum basilicum* contained significant amounts of Cd, Pb, and Zn. Instead, *Lavandula spica* contained significant amounts of Pb, Cd, and Zn in their leaves. Bai et al. [57] indicated that *Lavandula angustifolia* tended to accumulate metals mainly in its roots and stems. In another study, Dinu et al. [58] showed that *Ocimum basilicum* accumulated the heavy metals differently in plant organs. For example, in the roots system the Co(II), Pb(II), Cd(II) and Cr(III) were the main accumulated ions, while flowers accumulated Ni(II), Zn(II) and Cu(II). The concentration of Pb(II) and Cr(III) in roots were found to be over the maximum admissible limit. Fattahi et al. [20] found that in *Ocimum basilicum* L. the Pb(II) and Cd(II) levels were higher in leaves and roots than in the other components of the plant. Also, Cd(II) and Pb(II) levels were higher in leaves than in roots.

According to Asgari Lajayer et al. [59], *Ocimum basilicum* grown in soil containing Cu, Zn, Fe, Ni, Pb, and Cd, accumulated lower quantities of heavy metals in the aerial components than in the root when irradiated and non-irradiated sewage sludge were used as soil amendment. The results of the study conducted by Hashemi et al. [60] has shown that under Cd(II) stress *Lavandula stoechas* L. accumulated the ions in the aerial parts mainly in the leaf tissues. Another study using *Lavandula spica* L. showed that the root had a higher capacity to store cadmium than the other plant parts, the quantity accumulated increasing with Cd(II) level increase in soil [61]. More results concerning the Cd(II), Pb(II), Cu(II), Zn(II), Ni(II) and Mn(II) contents in medicinal plants parts harvested from different worldwide sites or from experimental studies are shown in Table 1.

## 4. Phytotherapy and Cosmetics—A Path for Human Health Risks Caused by Contaminated Medicinal Plants

**Figure 3:**
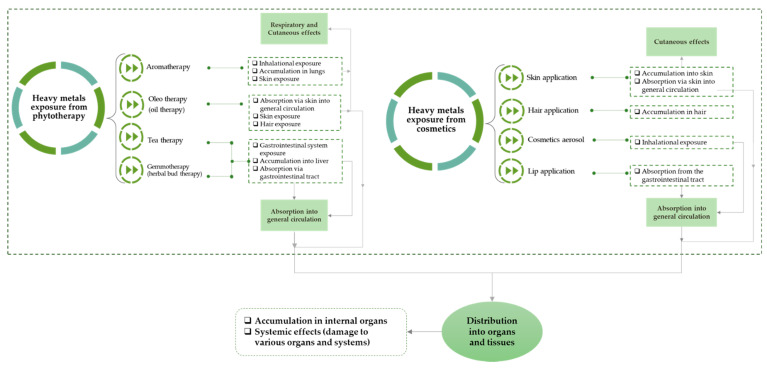
Human exposure to heavy metals from phytotherapy and cosmetics.

The personal care products/cosmetics sector is extremely competitive. Strong product brands and innovation determine the competitive position of the companies to change their strategies concerning risks to human health, in line with consumer requirements, so as to reduce heavy metals use included as pigments, preservatives, etc., ingredients that are no longer considered safe and have therefore been phased out by regulators. High concentrations of heavy metals in cosmetics may be due to the type and source of raw materials used, processing techniques, storage, and mode of transport. It is recognized that heavy metal impurities from personal care products are unavoidable due to the ubiquitous nature of these elements, but should be removed whenever technically is possible [83,84,85]. Figure 3 shows the pathways of human exposure to heavy metals from phytotherapy and cosmetics.

The routes of exposure of human body to heavy metals depends on the routes of penetration in organisms. The adverse effects are generated either immediately or after a long time period and have as result the absorption into the general circulation as a first step and the distribution into organs as tissues as the final step [86,87,88].

When plants are used for phytotherapy or cosmetic purposes, as represented in Figure 3, the external human exposure after the contact with a specific substance, results primarily in its penetration through the skin. When they are used in the oral cavity there is a significant systemic exposure and they may have effects on the face, lips, eyes and mucous membranes. Even for dermal contact or ingestion, the impact on the human body is the same; the presence of heavy metals in plants used for different natural treatments could cause accumulation in internal organs and induce the collapse of different organs (kidneys, heart, etc.) [83,85].

In the literature, there are some studies regarding the pathways into organism and their impact on human body. For example, Ullah et al. [89] investigated the level of heavy metals in cosmetics and their route exposure. They concluded that lipsticks, powder, shampoo, crema, as dermal exposure seems to be the most significant exposure route due to the direct contact with the skin. Łodyga-Chruścińska et al. [90] suggested that a greater attention should be paid for facial cosmetics, especially when in their composition are metals that can cause allergic contact dermatitis. They performed a survey of the metallic impurities in several lipsticks and face powders and the effect of long-term exposure to heavy metals. Shaban et al. [91] studied the levels of heavy metals in medicinal plant due to their use as natural oxidants, and concluded that the growth of plants in a contaminated environment should be kept to a minimum, and the use of plants that are collected from these sites should be studied before manufacturing. The ingestion or dermal contact of such medicinal plants is harmful for the human body.

## 5. The Interactions between Heavy Metals and Medicinal Plants and the Importance of Their Antioxidant Activity

Annual world crop production falls by up to approx. 70% due to abiotic stress, according to estimates, and this is worrying judging by population growth. By polluting the environment in various ways, the stress on plants from abiotic factors is further increased [92,93]. In view of this situation, it is imperative to understand the mechanisms of action of these abiotic factors, especially heavy metals, in order to observe the defense mechanisms of medicinal plants in crops, and to take firm measures for crops with a high yield in terms of quality and quantity.

These stressors endanger plant tissues, ultimately jeopardizing their survival if the antioxidant mechanisms do not act directly in proportion to the intensity of abiotic stress to which the plant is subjected. Plants have complex mechanisms that they use for survival. For example, when the intensity of a stressor is high, plants resort to the active, genetically engineered mechanism of programmed cell death (PCD) in their desire to survive. The PCD-inducing pathway usually involves an rise in concentrations of reactive oxygen species (ROS) which are used as mediators of the stress signal [94,95,96].

The main secondary metabolites are classified into three groups: (i) phenolics (phenolic acids, lignin, coumarins, stilbenes, lignans, flavonoids and tannins); (ii) terpenes (carotenoids, plant volatiles, sterols and glycosides); and (iii) nitrogen-containing compounds (glucosinolates and alkaloids) [97]. Depending on the tolerance of the medicinal plant species and the nature of the metal ions, the responses of defense in tissues may vary. Usually, the accumulation of heavy metal ions starts in the soil through the roots, at which point signals are transmitted to the plant tissues responsible for producing substances that have the ability to counteract them. A number of metabolites are secreted from certain organs. For example, essential oils are secreted by epidermal trichomes and secretory cells in the epidermis. In addition to trichomes, lipophilic materials are also produced by specialized cells occurring within the plant body (e.g., idioblasts, laticifers or epithelial cells) [98,99,100].

These compounds are delivered to the roots in the form of root exudates, which enhance detoxification mechanisms in the root tissue [101]. At the tissue level of the stems, leaves, and upper parts of medicinal plants, stress caused by the accumulation of metal ions can be activated in different tissue sections, thereby stimulating the secretion or repression of the stress gene network, which leads to the production of functional cellular molecules in accordance with the intensity of abiotic stress caused by the presence of metal ions. This form of adaptation can be in the form of osmoprotectants, detoxifying enzymes, transporters, chaperones, and proteases that serve as the first line of cellular protection [102].

From germination to full maturity, plants are subject to two types of stress, namely biotic and abiotic, with effects varying in intensity and duration depending on a number of factors [103]. A wide range of microorganisms attack plants throughout their development, thus creating additional stress on top of that induced by lack of water, extreme temperatures, high concentrations of heavy metals, excessive light, UV radiation, pollutants (e.g., ozone and herbicides), excessively high salinity, and others [104,105].

**Figure 4:**
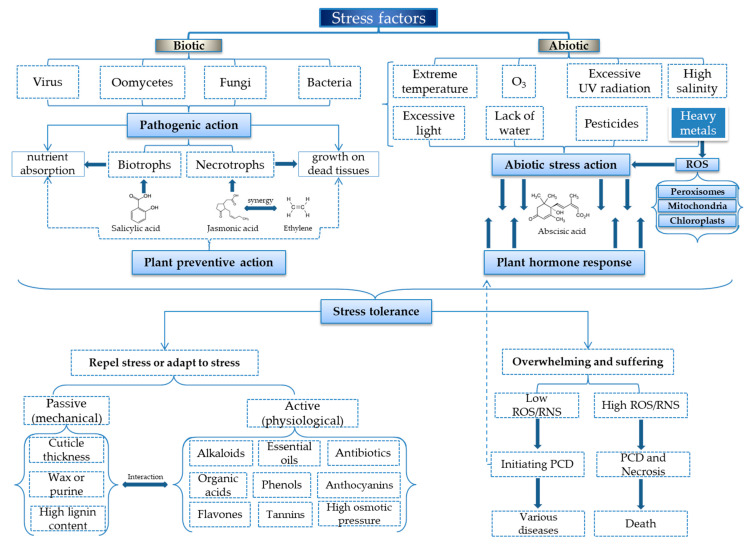
Schematic representation of stress factors, their action on plants/herbs and defense mechanisms.

Even if reactive oxygen species (ROS) have a high toxicity, in vivo, depending on their concentration, they can play a dual role: at low concentrations in the initial phase, ROS mediate at least part of the stress responses, stimulating the plant to use defense mechanisms, but in large quantities, ROS usually lead to stress, and if the stress persists, to PCD. This is why metalloproteins with antioxidant capacity are indispensable in maintaining the plant in optimal parameters [95,106]. Figure 4 shows the schematic representation of stress factors, their action on plants/herbs and defense mechanisms.

To better define how stress and the defense strategies work in plants, the main stressors are classified as biotic and abiotic. Throughout a plant’s life, it is constantly undergoing changes to which it must adapt in order to survive and reach maturity. Usually, these changes of whatever nature are biotic stressors (viruses, fungi, rust, bacteria, fungi, etc.) and abiotic stressors (extreme temperatures, too much or too little light, ozone, lack or low amounts of water, high salinity, pesticides, excessive UV radiation, and last but not least heavy metals) [107,108].

Regardless of the type of stress, plants will certainly slow down their growth and development, especially if their defensive mechanisms do not intervene in time, or do not cope with the stressor. These changes in plant growth and development can be plastic (when factors such as frost, lack of water, heavy metal action, high temperatures, etc. intervene), or elastic (e.g., when there is reduced light) [109]. The time in which stressors act on the plant varies from a few hours (in the case of temperature), to a few days (in the case of the amount of water in the soil), or more weeks (nutrient deficiencies) [110].

The biotic stress is described as stress produced by a living organism that damages the plant [108]. By feeding mode the biotic pests are: biotrophs and necrotrophs. Biotrophic pathogens leave the plant cell alive but absorb nutrients from its tissues. In response to their action, the plant often induces a PCD, thus causing the death of all cells in the vicinity of the pathogen, limiting its spread [111]. After the onset and occurrence of PCD, necrotrophs grow on the dead tissue. To respond to these two types of invasions, plants have developed two distinct methods. Resistance to biotrophic pathogens is due to salicylic acid, and resistance to necrotrophic pathogens is due to jasmonic acid and ethylene. These hormones play a role in plant growth and development. In the case of biotic stress, they interact synergistically and antagonistically [108,112,113].

If the balance between pathogens and their counteracting mechanisms is maintained, the plant does not undergo major changes. However, when abiotic factors also act on the plant, the plant inevitably suffers, since the focus is mainly on abiotic factors to the detriment of biotic ones. In biological systems, during metabolism, plants generate both reactive oxygen species (ROS) and reactive nitrogen species (RNS), which occur as normal physiological processes. However, in extreme environmental conditions (e.g., salinity, drought, floods, high temperature, heavy metals content etc.) the level of ROS and RNS production increases, and this produces a significant increase in oxidative stress [114]. ROS synthesis is especially prominent in subcellular organs such as peroxisomes, chloroplasts and mitochondria, the latter two being the preferred site for metal-induced ROS production, as ROS have a strong oxidative character [115].

Even though the two types of stress do not seem to be related, the response to both is given by increased ROS and abscisic acid (ABA), the level of this hormone increases during abiotic stress, especially under drought conditions [112,113]. It increases in direct proportion to the stress of biotic and abiotic factors, to the point where it establishes equilibrium or to the point where the plant cannot keep up with the stressors and an imbalance occurs between stress and plant response, at which point PDC mechanisms arise to counteract abiotic stressors and stagnate development alongside other mechanisms to counteract abiotic factors [108]. Abscisic acid (ABA) is a key hormone involved in adaptive responses to several types of abiotic stress and also has a remarkable impact on plant defense against various pathogens [112].

Medicinal plants have a number of mechanisms by which they respond to the action of abiotic factors, including heavy metals, as follows. First, they detect the stressors, then they translate the signal and transmit it to cell receptors. From this point, the phase of counteracting stressors begins through a multitude of mechanisms specific to the stressor, its intensity and the plant’s ability to counteract the stressor by modulating the physiological, biochemical and molecular state of the cell. Heavy metals are a stressor for medicinal plants. However, the damage caused varies according to the nature of the metal on the one hand and its quantity on the other hand, of course the ability of medicinal plants to react to these stressors also plays a role. The literature mentions a number of mechanisms by which heavy metals interfere with the normal functioning of medicinal plants, namely: (i) interference with functional sites in proteins; (ii) displacement of essential elements, thus disrupting enzyme functions; or (iii) increased ROS production above the limit where medicinal plants can counteract ROS effects without suffering. Redox-active metals such as Cu and Fe directly induce ROS production via Fenton and Haber-Weiss reactions [116].

At the cellular level, medicinal plants become vulnerable to abiotic stressors that are harmful to tissues and can affect plant productivity, growth or even survival. As mentioned, these stressors lead to the accumulation of ROS that interact with cell molecules and metabolites and can trigger irreversible metabolic abnormalities and cell necrosis [117,118]. Another effect of increased ROS production can lead to cell death by inducing the apoptosis signal by lipid peroxidation, inhibition of enzymatic activities, damage to nucleic acids, or oxidation of proteins [119].

In order to maintain the optimal level between ROS and the plants’ ability to counteract, it is necessary to highlight the importance of metalloenzymes which include an important group of proteins that have a metal ion cofactor in their composition. For survival and growth, plants have a number of physiological pathways in which these metalloproteins are also involved. ROS metabolism in plants involves a number of enzymes with a protective role against oxidative stress; these are metalloproteins which are some of the most important enzymes and have a number of key functions in cells such as acting as enzymes in plant metabolism, having transport and storage and signal transduction functions [120,121].

When heavy metals, both essential (Fe, Cu, etc.) and toxic (Ni, Pb, Cd, Hg, etc.), cause abiotic stress to plants, metalloenzymes intervene and partially or totally counteract this stress. However, when the stress induced by these heavy metals is high enough to cause imbalances in the redox mechanism, oxidative stress occurs, in which case the capacity of the plant’s antioxidant defense system is exceeded, and this inevitably leads to increased production of ROS. Regarding the occurrence of imbalance between stressors and the plant’s ability to counteract their effects, two possible situations arise, namely [97]:

(i) The plant suffers a series of abnormalities (when the amount of ROS is relatively low), in terms of growth, development, ripening and reproduction, initiating the phenomenon of PCD and necrosis, but still survives, or
(ii) In extreme cases when the amount of ROS is above the plant’s ability to counteract and the phenomenon of PCD and necrosis occurs, leading finally to the death of the plant.

The ability of plants to signal redox throughout the plant is remarkable, especially when the amount of ROS increases, it has the capacity to counteract the undesirable effects of the accumulation of excessive amounts of ROS [116]. In contaminated soil the heavy metals are absorbed into the plant and stress signals occur in the whole plant. These signals are then sent to the organs responsible for counteracting the stress and at the level of different cell compartments substances with antioxidant character are produced to act locally against ROS [99,116]. Some heavy metals such as Cd, Pb, and Zn, which belong to the category of non-redox active metals, stimulate the increase of ROS content only by indirect mechanisms. An example of indirect stimulation of ROS production is the inhibition of enzymes that function in the cellular antioxidant defense network [116].

As previously presented, in medicinal plants, ROS accumulation depends on the balance between ROS production and ROS scavenging [122], which in turn also depends on growth conditions such as soil salinity, lack or excess of water, temperature extremes, light intensity, presence of pesticides or heavy metals in the soil, etc. Another undesirable effect of heavy metals is the limitation of CO_2_ fixation in chloroplasts, which, together with an over-reduction of the electron transport chain involved in the photosynthesis process, is a major site of ROS production [122].

The mechanisms by which heavy metals act on medicinal plants are varied and with toxic effects, some of the most common being: (i) competition in uptake at the root surface with certain similar nutrient cations required for plant growth (for example, As and Cd compete with P and Zn for uptake respectively); (ii) inactivation of functional proteins by direct interaction of heavy metals with the sulfhydryl (-SH) group of functional proteins, resulting in inactivation by disruption of their structure and function; (iii) migration of essential cations from specific binding sites due to heavy metals, leading to collapse of function; and last but not least, (iv) generation of ROS [123,124].

As presented so far, plants that are affected by oxidative stress caused by heavy metal pollution, in the framework of ROS metabolism, need the presence of metalloenzymes with antioxidant character to maintain their characteristics at an optimal level of their growth and development. The most representative metalloenzymes are catalase (CAT), superoxide dismutase (SOD), ascorbate peroxidase (APX), and xanthine oxidoreductase (XOR). In the following we will also briefly discuss how they act to counteract the effects of ROS metabolism on plant growth [120,121].

**Figure 5:**
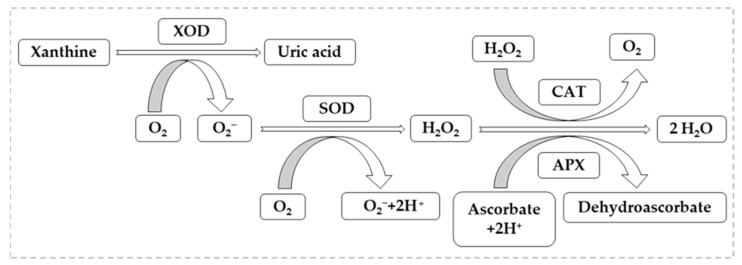
Main reactions of metalloenzymes involved in ROS metabolism in plant cells.

As mentioned above, a number of microelements are indispensable to living organisms, in low doses, when these exceed the limit of necessity for plants, they become a factor of oxidative stress [125,126]. For example, Cu helps normal plant growth and development by being a cofactor in the formation of copper zinc-superoxide dismutase (CuZn-SOD), but if the limits are high, it becomes a stress factor causing multiple toxic effects in plants [127]. In the case of Fe, as in the case of Cu, its absence leads to nutritional disorders for many dicotyledonous species growing in alkaline soils, where its availability is reduced. Fe is an indispensable element for normal plant development, entering the metabolism of plants requiring redox exchange [128]. Being a widespread metal in many soil types, where it is present in large quantities, it produces phytotoxic effects [129]. Figure 5 shows the mode of action of oxidative stress factors represented by XOR, in this case it is the source of ROS (mainly superoxide radicals), and the synergistic action between metalloenzymes CAT, SOD and APX with antioxidant character, counteracting stress conditions [130].

Analyzing Figure 5, we can highlight the importance of metalloenzymes involved in ROS metabolism, one of the most important enzymes that is also used as a maker in cell biology and biochemistry being CAT, characteristic of peroxisomes. Its importance lies in its ability to remove H_2_O_2_ regardless of how it is present, either by its own metabolism or by accumulation [131,132].

To alleviate heavy metal-induced oxidative damage, inside the plant cells antioxidant enzymes as well as non-enzymatic antioxidant compounds are produced and thus the ROS-scavenging machinery is activated. To cope with the toxicity of metal ions accumulated into the cytosol through chelation by complexation with ligands, the metals bioavailability to plants is reduced. The organic acids, amino acids, phytochelatins, metallothioneins, cell wall proteins/pectins/polyphenols are secreted by plants and involved in heavy metal ion chelation [133,134,135]. Georgiadou et al. [136] observed that along with the increase of heavy metals concentration in soil (Ni, Cu, Zn), the nitro oxidative response and malondialdehyde (MDA) content in *Ocimum basilicum* L. leaves were raising up. CAT, SOD, and APX are strongly related with hydrogen peroxide content (H_2_O_2_). In comparison with control samples, it was observed that chlorophylls, anthocyanins and carotenoids decreased along with the increase of heavy metals concentration. Similar results were observed by Santos et al. [137] in *Lavandula penduculata* grown in soils contaminated with Al, As, Ca, Cd, Cr, Cu, Fe, K, Mg, Mn, Mo, Ni, Pb, Sb, V, and Zn. More research results on the effects of heavy metals levels variation in the growth systems on the enzymatic and non-enzymatic antioxidants in some medicinal plants are summarized in Table 2.

## 6. Assessment of Risks to Human Health

Health risk assessment of heavy metal toxicity implies determination of the probability of an adverse event at a certain level of exposure. Risk analyses are assessed both for chronic and acute exposures following the environmental or occupational exposure, but the chronic exposure is most often assessed. In the assessment of short-term exposure to heavy metals or other toxic substances, the emergency room physicians or poison control centers who diagnose, provide treatment, manage poisoning and assist in preventing further exposure are normally involved. The data collected by poison centers are then delivered to public health agencies and regulatory institutions which are concerned about lifetime risks [162,163].

The Food and Drug Administration, the Environmental Protection Agency, Toxic Substances Agency and Disease Registry, and Occupational Health and Safety National Institutes are well-known agencies whose mission is to assess the chronic exposure to a hazardous material or substance. International agencies, such as the World Health Organization’s International Program on Chemical Safety and the International Labor Agency, provide guidance to member countries [162]. The Joint FAO/WHO Expert Committee on Food Additives (JECFA) is the international scientific expert committee that serves as a scientific advisory body to WHO Member States on the safety of food additives, residues of veterinary medicinal products in food stuff, natural toxic substances and contaminants in food stuffs, including metals [162,164]. The methodologies followed by these agencies result in a general agreement on health risks, but in the current regulatory policies the social and political policies are the main decision-makers [162,164]. EPA’s Integrated Risk Information System (IRA) program, operated by the U.S. Environmental Protection Agency, provides the required information for human health protection through risk assessment and management [162,164].

The risk assessment process is described as “*the characterization of potential adverse health effects of human exposure to environmental hazards*” [162]. Estimation of the health risk, whether present or potential, is based on the determination of the extent to which a group of persons has been or may be exposed to environmental hazards and on the degree of exposure given by the type and degree of hazard posed by the chemical substance. Risk assessment generally involves four steps: hazard identification, exposure assessment, toxicity assessment and risk characterization [162,165,166] and can be expressed as in Equation (1) [165].

(1) $$Risk=f\left(toxicity,\;exposure\right)$$

The exposure assessment determines the potential health effects of toxic effects usually expressed as the reference dose (RfD) which may result from excessive exposure to a metal. These are followed by dose-response studies, performed by epidemiological studies on human populations or based on animal studies. Human populations are rarely available (there are notable exceptions for lead, methyl mercury and arsenic) and consequently the initial stages of the risk assessment process involve laboratory animals [162]. These studies determined the no-observed-adverse-effect level (NOAEL) and the lowest-observed-adverse-effect level (LOAEL). The RfD values derives from NOAEL reported to uncertainty factors (UF, MF) that reflect different types of data used for RfD estimation (Equation (2)) [162,167].

Although various empirical studies on humans were performed, there are still uncertainties due to continuous changes of lifestyle or variations in biology. Therefore, in contaminated areas, the use of toxicokinetic/pharmacodynamic risk assessment or predictive assessments models become increasingly necessary. Predictive models of risk assessment include a number of physiological or biological variables. It is necessary to take into account the differences between the mechanisms for different metals and metal compounds and the variables of human sensitivity to certain metals [162,165,168].

Heavy metals and their compounds can pose negative effects in any human organ or on physiological system, the nature and severity of toxicity depending on the metal involved, chemical and valence states, duration of exposure (acute or chronic), exposure level and the age of the exposed person [169]. Children and young people are especially vulnerable to heavy metal intoxication [169,170]. Through target organs or end organs the effects can be identified. According to EPA IRIS program “*the effect on the target organ may be the critical effect, or the first adverse effect, or its known precursor, that occurs in the most sensitive species as the dosage rate of an agent*” [165]. The target organ mostly damaged by exposure to heavy metals include the central and peripheral nervous system, cardiovascular, renal, hematopoietic, gastrointestinal, musculoskeletal, immunological, and integumentary systems [165,169].

EPA uses risk assessment to characterize the nature and extent of risks to human health (e.g., residents, workers, etc.) and environmental receptors (birds, fish, wildlife) that come from chemical contaminants and other stressors, which may be present in the environment [171].

Risk assessment is, as far as possible, a scientific process. In general, the risk depends on the following three factors: (i) the amount of chemical present in an environmental ecosystem (e.g., soil, water, air); (ii) the level of exposure that a person or ecological receptor may have with the contaminated environmental factor; (iii) the inherent toxicity of the chemical product/species [171].

Based on the information gathered in the planning and delimitation phase, the risk assessor estimates the frequency and extent of human and environmental exposures that may occur as a result of contact with the contaminated environment, both now and in the future. This exposure assessment is then combined with information on the inherent toxicity of the chemical substance (the expected response at a given level of exposure) to predict the probability, nature and extent of adverse health effects that may occur [165,172]. After the identification of the uncertainties from risk estimation and characterization of the real reliability (or lack of reliability) of the resulting risk estimates, risk managers take the final decision on protecting the natural environment and humans from the so-called stressors [165].

It should be emphasized that “*risk managers*” can be state or federal officials whose role is to protect the environment, business leaders who work in companies and who can affect the environment or private citizens who make decisions about risks [165,171].

Table 3 shows the equations included in the risk assessments of heavy metals in food products, while Table 4 focuses on identifying the hazard quotients (HQ) and the hazard index (HI) of heavy metals in the medicinal plant or associated products. The coefficients values greater than 1 reveal if the exposure could indicate adverse effects [12,23].

The intake of herbal food-based dishes and herbal products such as teas or cosmetics is carried out without clear consumption guidelines to avoid possible toxic effects due to ingestion of heavy metals or other chemicals. In a hypothetical scenario involving long-term consumption of medicinal plants, all of the plants surveyed fell within the acceptable limits that may involve risks to human health.

## 7. Conclusions

Exposure of medicinal plants to various concentrations of heavy metals triggers some stress strategies to alleviate physiological and biochemical responses in cells. Our investigation revealed that the accumulation of heavy metals in medicinal plant tissues varies based on plant species and metal elements. Different detoxifying mechanisms are involved in heavy metals absorption, translocation and compartmentation based on the pollution sources and the exposure pathways. Unfortunately, these mechanisms still require investigation since they are poorly understood. The ROS-scavenging machinery is activated with the increase or decrease of the antioxidant enzymes and non-enzymatic antioxidants due to the heavy metal induced stress. On the other side, when heavy metals enter the food chain through medicinal plants or associated products consumption, their excessive bioaccumulation could lead to numerous health issues.

Our survey showed that the concentration of heavy metals detected in different medicinal plants or associated products provides values of the HQ or HI lower than 1, suggesting that the risks to human health are in an acceptable limit. The unavailability of regulatory provisions in the field of medicinal plants and their unrestricted and unlimited use in various preparations can lead to risks for consumers, making it essential to comply with quality control guidelines and to establish safe levels of heavy metal concentrations that do not cause risks. Overall, cultivation of medicinal plants or collection of their edible parts from possible heavy metals contaminated areas should be avoided due to their well-known risks to human health.

## Figures and Tables

**Table 1:** Heavy metals contents in medicinal plants compartments.

Plant sp.	Plant Samples Origin	Plant Part	Heavy Metals Ions Concentration (mg/kg)						Refs.
			Cd(II)	Pb(II)	Cu(II)	Zn(II)	Ni(II)	Mn(II)	
*Oenothera* *biennis*	Qingchengzi mining area, China	roots	0.15–1.22	43–83.2	1.7–3.8	43.9–212.7	-	-	[62]
		aerial parts	0.12–0.79	5.5–42.4	1.6–2.9	36.6–151.8	-	-	
*Taraxacum* *mongolicum*		roots	0.93–2.20	128.4–212.2	7.3–8.9	35.9–127.2	-	-	
		aerial parts	2.11–5.18	51.7–71.7	5.8–11.5	23.1–58.2	-	-	
*Plantago asiatica*		roots	0.31–15.0	65.9–344.7	14–18.4	90.1–651	-	-	
		aerial parts	0.30–14.5	20.9–181.8	7.6–17	70–344.9	-	-	
*Portulaca oleracea*		roots	0.83	25.8	11.7	129.8	-	-	
		aerial parts	0.37	21.8	9.9	73.1	-	-	
*Echinacea pallida* var. pallida	Laboratory experiments in greenhouse	roots	0.13–0.15	2.305–2.989	15.164–18.420	28.787–39.604	3.689–5.983	81.35–97.37	[63]
		aerial parts	0.10–0.11	0.412–0.753	8.979–22.241	14.463–14.957	0.898–0.983	8.91–9.25	
*Echinacea* *purpurea* var. purpurea		roots	0.11–0.24	1.770–7.247	25.326–25.284	51.72–142.23	3.307–16.155	60.41–272.78	
		aerial parts	0.10–0.12	0.345–1.015	17.194–27.875	21.445–22.577	1.229–2.485	24.80–25.21	
*Echinacea* *purpurea* var. baby white swan		roots	0.12–0.13	1.198–2.211	16.459–29.079	23.569–27.267	3.625–4.481	54.50–70.81	
		aerial parts	0.08–0.11	0.648–0.834	10.453–12.709	18.798–19.370	1.208–2.399	16.54–32.05	
*Echinacea* *purpurea* var. double decker		roots	0.098–0.10	0.481–1.818	8.495–17.822	23.755–23.789	0.595–3.412	41.27–57.97	
		aerial parts	0.103–0.11	0.390–0.793	10.350–14.509	16.278–19.621	1.445–1.837	19.66–41.13	
*Echinacea* *paradoxa* var. paradoxa		roots	0.116–0.13	0.286–1.893	6.173–35.347	21.178–32.894	0.716–3.927	33.65–66.64	
		aerial parts	0.09–0.12	0.551–2.180	10.397–23.762	17.441–26.995	1.052–4.821	13.67–78.67	
*Lavandula* *vera* L.	Agricultural fields contaminated by the Non-Ferrous-Metal Works, KCM- Plovdiv, Bulgaria	roots	2.3–160.9	20.5–1566.9	-	46.8–1755.9	-	-	[64]
		stems	3.1–27.1	71.2–2157.9	-	74.9–683.2	-	-	
		leaves	28.4–113.2	89.97–5784.7	-	80.2–2881.9	-	-	
		flowering stalks	0.62–15.6	31.4–1147.3	-	57.1–349.3	-	-	
*Matricaria* *chamomilla*	Commercially available teas produced in different European Countries	flowers	0.08–0.81	0.56–1.28	4.09–11.4	63.4–109	-	54.7–184	[65]
*Camellia sinesis*		leaves	36.3–202	1.50–4.77	9.35–22.6	30.7–116	-	1.15–1.76	
*Achillea* *millefolium*	From spontaneous flora of Galați County, Romania and different indigenous products (herbal teas packs)	flowers	0.02–0.04	0.09–0.42	11.00–15.00	-	-	-	[66]
*Calendula* *officinalis*		flowers	0.01	0.14–0.24	4.00–24.00	-	-	-	
*Matricaria* *chamomilla*		flowers	0.01–0.02	0.04–0.49	11.00–17.00	-	-	-	
*Ocimum* *basilicum*		aerial parts	0.01	0.60–2.03	10.00–15.00	-	-	-	
*Tilia cordata*		flowers	0.01–0.05	0.01–0.32	0.00–17.00	-	-	-	
*Anethum* *graveolens*		leaves	0.02–0.12	0.08–0.09	127–142	-	-	-	
*Origanum* *majorana*		aerial parts	0.05	0.14–0.95	131–348	-	-	-	
*Origanum* *vulgare*		aerial parts	0.06–0.07	0.04	198–201	-	-	-	
*Mentha piperita*		leaves	0.01–0.10	0.18–1.28	157–309	-	-	-	
*Petroselinum crispum*		leaves	0.05–0.07	0.06–0.11	122–133	-	-	-	
*Thymus vulgaris*		aerial parts	0.03–0.09	0.07–0.11	131–191	-	-	-	
*Thymus Vulgaris*	Ash-shouback south region, Jordan	aerial parts	BLD	33.03	13.23	16.18	23.85	15.52	[67]
*Tymus* *serpyllum*		aerial parts	BLD	1.26	10.40	15.80	BLD	14.7	
*Saliva* *officinalis*		aerial parts	BLD	BLD	7.66	114.91	0.47	44.0	
*Hypericum* *perforatum*	Site close to a country road inside the Canale Monterano Natural Park/Site located in the inner city of Rome, Italy	flowers	0.03/0.27	2.88/3.96	6.27/9.36	21.33/26.46	0.40/5.80	-	[68]
		roots	0.02/0.77	3.40/73.30	6.45/8.75	29.82/25.60	0.04/1.12	-	
		cauline leaves	0.02/0.72	0.24/7.82	2.71/10.13	20.66/29.33	0.32/1.32	-	
		stalk	0.02/0.27	0.02/70.30	1.09/2.25	18.10/26.70	0.02/0.09	-	
*Dactylis* *glomerata*		flowers	0.09/0.02	0.47/1.55	1.77/2.75	10.45/8.47	0.09/0.02	-	
		roots	0.76/0.81	0.35/30.32	3.97/6.76	14.91/39.17	0.04/0.05	-	
		basal leaves	0.34/0.96	0.74/8.60	0.98/5.28	9.14/17.20	0.39/0.36	-	
		stalk	0.43/0.39	0.13/0.61	0.36/2.00	6.76/9.24	0.02/0.91	-	
*Plantago* *lanceolata*		flowers	0.02/3.12	0.59/1.89	4.20/0.12	37.42/18.87	0.02/3.45	-	
		roots	0.12/0.84	0.52/8.48	7.80/17.38	14.97/67.13	0.19/0.44	-	
		cauline leaves	0.01/0.01	0.77/2.91	1.93/4.94	6.35/20.25	0.39/0.02	-	
		stalk	0.02/0.99	0.04/2.10	0.79/2.96	2.67/13.38	0.02/0.06	-	
*Verbascum* *thapsus*		flowers	0.07/0.02	0.10/0.83	1.21/6.20	2.12/19.91	0.11/0.50	-	
		roots	0.05/0.08	1.83/3.50	7.48/9.24	3.72/36.88	0.53/0.02	-	
		cauline leaves	0.02/0.15	0.56/8.81	3.79/8.30	3.60/27.31	0.17/0.29	-	
		stalk	0.02/0.02	0.26/9.21	3.27/9.24	1.49/15.60	0.02/0.02	-	
*Cichorium* *intybus*		flowers	3.51/0.05	0.11/0.58	3.48/4.81	18.75/15.29	1.11/0.79	-	
		roots	0.17/0.19	0.40/4.82	3.67/7.86	11.77/17.7	0.49/0.50	-	
		basal leaves	0.02/1.16	0.29/4.81	1.76/21.25	23.27/53.90	0.02/1.16	-	
		cauline leaves	0.02/0.27	BLD	2.59/6.40	3.31/66.35	0.45/0.33	-	
		stalk	0.15/0.11	0.02/4.32	2.29/2.84	5.34/35.75	0.33/0.19		
*Hypericum* *perforatum*	“Dealul Bujorului” Natural Reservation and “Izvorul Cerbului” camping, Romania	flowers	0.16–0.68	-	22.56–43.62	23.66–30.64	-	11.91–20.67	[69]
		leaves	0.078–0.84	-	0.465–5.602	30.2–75.62	-	194.6–254.2	
		stems	0.42–4.65	-	17.64–19.85	13.16–17.64	-	7.25–15.74	
*Hypericum* *perforatum*	Sites from the Rhodope Mountains	aerial parts	0.35–0.92	0.7–1.9	5.6–9.1	21–47	0.7–11.7	12–69	[70]
*Valeriana* *officinalis* L.	Soil from agricultural land located away from excessive traffic, Krokocice village, Poland	roots	-	-	8.50	49.5	-	81.0	[71]
*Cichorium* *intybus* L.	Van Lake Basin Van Turkey	aerial parts	0.15	0.14	10.80	18.84	-	20.04	[72]
*Urtica* *dioica*	Meža Valley (Slovenia)	aerial parts	0.2–0.9	1.1–15.5	5.0–18.7	29.1–73.5	-	17.3–318.0	[73]
*Hypericum* *perforatum*		aerial parts	0.2–4.2	2.1–25.1	6.3–11.7	26.9–103.4	-	7.8–75.4	
*Achillea* *millefolium*		aerial parts	0.3–5.7	1.5–15.9	4.2–9.9	23.2–211.7	-	16.6–163.2	
*Plantago* *lanceolata*		aerial parts	0.3–16	1.4–195.9	4.2–9.3	33.3–799.5	-	10.3–75.4	
*Thymus* *serpyllum* L.	Kuyavia- Pomerania Province, Poland	inflorescences	-	4.1–6.80	21.75–30.85	64.75–106.2	-	124.3–338.6	[74]
		leaves + stems	-	12.00–17.90	7.80–16.20	41.95–116.9	-	128.0–289.1	
		roots	-	5.00–20.80	22.00–28.20	34.05–109.4	-	119.6–264.6	
*Urtica* *dioica*	Sites in Spreča river valley, Bosnia and Herzegovina	aerial parts	0.022	0.04	13.81	31.8	1.7	-	[75]
*Artemisia* *vulgaris*		aerial parts	0.034	3.36	16.92	34.5	42.9	-	
*Mentha* *arvensis*		aerial parts	0.019	1.31	13.87	32.2	7.6	-	
*Urtica urens*		aerial parts	BLD	BLD	12.9	38.9	5.2	-	
*Achillea* *millefolium*		aerial parts	BLD	BLD	12.1	36.2	6.1	-	
*Calendula* *officinalis*	Natural habitats of the plants, region of Southeast Serbia	flower	-	-	12.82	18.15	-	24.38	[76]
*Primula* *officinalis*		flower	-	-	20.35	22.36	-	36.60	
*Origanum* *vulgare*		aerial parts	-	-	23.95	49.65	-	21.80	
*Cichorium* *intybus*		aerial parts	-	-	20.50	32.40	-	49.39	
*Saturea* *montana*		aerial parts	-	-	15.77	25.12	-	40.65	
*Delphinidum consolida*		aerial parts	-	-	23.06	39.84	-	13.84	
*Papaver* *rhoeas*		flower	-	-	35.50	31.80	-	20.61	
*Atropa* *belladonna*	Laboratory studies	seeds	0.11	BLD	11.61	50.58	1.13	18.81	[77]
		roots	0.14	BLD	7.44	15.03	2.89	17.56	
*Tanacetum* *vulgare* L.	Landfill site located in the Pilsen Region, Czech Republic	root	0.294–1.63	1.335–18.03	29.35–75.82	39.50–166.2	8.84–77.66	138.0–560.7	[78]
		stalk	0.620–2.077	0.029–4.141	3.331–33.85	40.82–230.6	0.241–70.65	38.10–237.2	
		leaf	0.609–2.190	0.639–6.240	17.91–62.86	58.30–721.9	6.77–163.5	172– 401.1	
*Hypoxis helmerocallidea*	Muthi shops and open street markets in Pietermaritzburg KwaZulu-Natal, South Africa	tuber	0–0.04	0.58–1.50	7.83–11.90	27.15–40.67	2.54–2.59	182.25–234.79	[79]
*Rapenea* *melanophloeos*		stem bark	0–0.04	0.24–1.96	1.96–3.47	3.99–5.16	2.47–2.52	55.14– 163	
*Bulbine* *natalensis*		root	0.24–0.28	3.69–5.16	4.58–19.70	51.68–107.33	3.82–6.28	169– 479	
*Alepidea amatymbica*		root	0.27–0.39	0.37–4.54	13.00–19.33	45.25–55.93	12–30	238–248.69	
*Drimia* *elata*		bulb	0.01–0.06	0.22–1.23	5.61–11.25	34.07–102.57	4.24–10	60.69–145.76	
*Lycopodium clavatum*		whole plant	0.11–0.15	0–3.93	6.25–7.15	20.48–24.37	4.56–6.55	144– 315	
*Schizocarphus nervosus*		bulb	0–0.01	0.1–0.42	2.46–3.71	19.79–25.28	398–4.89	41.86–49.57	
*Momordica* *foetida*		root	0.01	2.87–3.62	5.78–6.61	34.56–36.11	5.78–6.61	44.77–55.28	
*Carduus* *nutans*	Historical polluted sites from Navodari area, Romania	leaf	0.005–0.053	2.18–25.63	1.88–15.21	12.66–31.01	0.211–1.29	-	[80]
*Taraxacum* *officinale*		leaf	0.002–0.078	1.117–13.79	5.70–13.84	20.45–35.49	0.108–0.812	-	
*Maximum permissible levels (mg/kg)*			0.3	10	20	50	-	-	[81,82]

BLD—below limit of detection.

**Table 2:** Heavy metals effects on enzymatic and non-enzymatic antioxidants in medicinal plants.

Plant	Metals Ions	Level of Heavy Metals	Plant Growth System	Plant Component	Enzymatic Antioxidant	Nonenzymatic Antioxidant	Refs.
*Camellia* *sinensis*	Cu(II)	50–600 µM	hydroponic culture	root and leaves	SOD↑ POD↑ CAT↑ APX↑	MDA↑ Phenol↑	[138]
*Erigeron* *annuus*	Cd(II)	0–200 µM	hydroponic culture	root, stem, and leaves	SOD↓ CAT↓ POD↑	MDA↑ Proline↑ NPT↑ GSH↑ PC↑	[139]
*Mentha* *spicata*	Cr(III) Cd(II) Co(II) Ni(II) Pb(II)	0–30,512 µg/g 1.2–100 µg/g 1.1–45 µg/g 0–60 µg/g 0–38 µg/g	soil and sludge	root, stem, and leaves	SOD↑ POD↑ CAT↑	MDA↑ Proline↑	[140]
*Solanum* *nigrum*	Cr(III)	0, 0.5, 1 mM	soil	leaves	SOD↑ POD↑	Proline↑	[141]
				roots	-	Citric acid↑ Malic acid↑	
*Parthenium* *hysterophorus*			soil	leaves	SOD↑ POD↑	Proline↑	
				root	-	Citric acid↑ Glutamic acid↑ Malic acid↑	
*Urtica* *dioica*	Cd(II)	0, 0.045, and 0.09 mM	hydroponic culture	root, stem and leaves	GR↑ GST↑ GSH-Px↑	GSH↑ GSSG↑ LPO↑	[142]
*Withania* *somnifera*	Fe(II)	25, 50, 100 and 200 µM	hydroponic culture	root and leaves	SOD↑ CAT↑ GPX↑	-	[143]
*Lemna* *minor*	Co(II)	0, 0.01 and 1 mM	hydroponic culture	plants	SOD↓	TBARS↑	[144]
*Nicotiana* *tabacum*	Cd	0, 100 and 500 µM	hydroponic culture	plants	SOD↑ APX↑ GPX↑ CAT↑	Proline↑ GSH↑ GSSG↑	[145]
*Coriandrum* *sativum* L.	Pb	0, 500, 1000, 1500 mg/kg	soil	plants	CAT↑ POD↑ SOD↑	Flavonoid↑ Vitamin C↓ MDA↑	[146]
*Mentha* *piperita*	Ni(II)	100, 250, and 500 μM	hydroponic system	roots and leaves	CAT↑ APX↑ POD↑ SOD↑	Soluble proteins MDA↑ Carotenoids↓ H_2_O_2_↑ protein ↓	[147]
*Ocimum* *basilicum* L.	Cd(II) and Al(III)	0–100 mg/kg	soil	epigeal parts	DPPH↑	Phenols↑ Flavonoids↑ flavanols↑	[148]
*Matricaria* *chamomilla*	Mn(II)	0 and 1000 μM	soil	shoots	APX↑ GPX↑ CAT↑ GR↓	AsA ↓ NPT↓ Soluble proteins↓ Soluble phenols↓	[149]
*Mentha* *piperita* L.	Cd(II)	0–40 mg/kg	soil	leaves	CAT↑ APX↑ PPO↑	Proline↑ MDA↑ Total Phenol↑ Total protein↓ H_2_O_2_↑	[150]
*Hypericum* *perforatum*	Cd(II)	0, 10 μM	hydroponic	shoots	PAL↓	Total soluble phenols↑ Flavonols↑ Epicatechin↑ Procyanidins↑ Proline↑ MDA↑ Ascorbic acid↓ Glycine↓ GSH↓ GSSG↓	[151]
				root	PAL↓	Total soluble phenols↑ Flavonols↓ Epicatechin↑ Procyanidins↓	
*Origanum* *vulgare* L.	Ni(II)	0–500 ppm	soil mixed with perlite	leaves	-	Anthocyanins↓ Carotenoids↓ MDA↑ Proline↑ Total Phenols↑	[152]
	Cu(II)	0–1000 ppm	soil mixed with perlite	leaves	-	Anthocyanins↓ Carotenoids↓ MDA↑ Proline↑ Total Phenols↓	
	Zn(II)	0–3000 ppm	soil mixed with perlite	leaves	-	Anthocyanins↓ Carotenoids↓ MDA↑ Proline↑ Total Phenols↓	
*Matricaria* *chamomilla*	Cd(II)	0–360 μM	hydroponic culture	flowers	SOD↑ POD↑	MDA↑ Apigenin↑	[153]
*Matricaria* *chamomilla*	Zn(II)	43.2–343.2 mg/kg	orthic luvisol	anthodia	-	Apigenin↑ Herniarin↓	[154]
*Matricaria* *chamomilla*	Pb(II)	0–75 μM	hydroponic culture	leaves	-	Proline↑	[155]
*Ocimum* *basilicum*	Ni(II)	0–500 ppm	soil mixed with perlite	leaves	SOD↑ CAT↓ APX↑ NR↑ p5CS↑	Anthocyanins↓ Carotenoids↓ MDA↑ H_2_O_2_↑ NO↑ Proline↑ Protein↑ Profilin↓	[136]
	Cu(II)	0–1000 ppm	soil mixed with perlite	leaves	SOD↑ CAT↑ APX↑ NR↑ p5CS↑	Anthocyanins↓ Carotenoids↓ MDA↑ H_2_O_2_↑ NO↑ Proline↑ Protein↓ Profilin↑	
	Zn(II)	0–3000 ppm	soil mixed with perlite	leaves	SOD↑ CAT↑ APX↑ NR↑ p5CS↑	Anthocyanins↓ Carotenoids↓ MDA↓ H_2_O_2_↑ NO↑ Proline↑ Protein↓ Profilin↓	
*Matricaria* *chamomilla* L.	Cd(II)	0–40 mg/kg	soil	leaves	SOD↑ CAT↑	Carotenoids↑ Proline↑ Sugar↓ lipid peroxidation↑	[156]
	Pb(II)	0–180 mg/kg	soil	leaves	SOD↑ CAT↑	Carotenoids↑ Proline↑ Sugar↓ lipid peroxidation↑	
*Ocimum* *basilicum* L.	Cd(II), Pb(II) and Zn(II)	0.25, 16 and 46.03 mg/kg vs. and 14, 142 and 207 mg/kg	soil	shoots	CAT↑ GPX↑ GST↑ GR↑ GPO↑ MDHAR↓ DHAR↓ APX↓ PAL↑	MDA↑ H_2_O_2_↓ GSH↓ GSSG↓ ASC↓ DHASC↑ phenols ↑ flavonoids↑	[157]
*Origanum* *vulgare* L.	Cd(II), Pb(II) and Zn(II)	0.25, 16 and 46.03 mg/kg vs. and 14, 142 and 207 mg/kg	soil	shoots	CAT↓ GPX↑ GST↑ GR↑ GPO↑ MDHAR↓ DHAR↓ APX↑ PAL↓	MDA↓ H_2_O_2_↓ GSH↑ GSSG↓ ASC↑ DHASC↑ phenols ↑ flavonoids↓	
*Polygonatum* *sibiricum*	Cd(II)	0–54.60 mg/kg	soil	roots and aerial parts	SOD↑ POD↓ CAT↓	Polysaccharide↑	[158]
*Plantago* *lanceolata*	Cd(II) Zn(II) Pb(II) Cu(II) Mn(II)	2.7–301.2 mg/kg 358.8–70445.8 mg/kg 123.1–4230.9 mg/kg 12.9–74.1 mg/kg 64.7–779.2 mg/kg	metalliferous and non-metalliferous soil	leaves	POD↑ SOD↑	GSH↑ Proline↓	[159]
*Salvia* *officinalis*	Pb(II)	0–400 μM	hydroponic culture	leaves	APX↑ GPX↑ SOD↑ GR↑	Protein↓ MDA↑ H_2_O_2_↑	[160]
*Lonicera* *japonica*	Cd(II)	0–200 mg/kg	soil	leaves	APX↑ DHAR↑ MDHAR↑ GR↑	H_2_O_2_↑ GSH↑ GSSG↓ NPT↑ Proline↑	[161]

Enzymatic antioxidants: SOD—Superoxide dismutase; POD—peroxidase; CAT—Catalase; APX—ascorbate peroxidase; GPX—Guaiacol peroxidase; GR—glutathione reductase; DPPH—radical scavenging activity; GST—Glutathione S-transferase; GPO—Guaiacol peroxidase; MDHAR—Monodehydroascorbate reductase; DHAR—dehydroascorbate reductase; PAL—Phenylalanine ammonia-lyase activity; PPO—Polyphenol oxidase; GSH-Px—Glutathione peroxidase; NR—Nitrate reductase; p5CS—Pyrroline-5-carboxylate synthase. Non-enzymatic antioxidants: AsA—Ascorbic acid, NPT—Non-protein thiols; MDA: Malondialdehyde; H_2_O_2_—Hydrogen peroxide; GSH—Reduced glutathione; GSSG—Oxidized glutathione; ASC—Ascorbate; DHASC—Dehydroascorbate; TBARS—Thiobarbituric acid reactive substances; LPO—Lipid peroxidation; NO—Nitrite-derived nitric oxide; PC—Phytochelatins; ↑—increase; ↓—decrease.

**Table 3:** Equations used in risk assessments of heavy metals in food products.

Risk Equations	Equation Form		Equation Parameters	Refs.
Reference Dose, RfD	$RfD=\frac{NOAEL}{UF\times MF}$	(2)	*RfD* = Reference dose (mg/kg/day); *NOAEL* = No observed adverse effects level; *UF* = Uncertainty Factors; *MF =* Modification Factors.	[167]
Potential Dose, PD	*PD = C* × *IR*	(3)	*C* = Concentration (mg/kg); *IR* = Ingestion rate (g/day).	[173]
Average Daily Dose, ADD	$ADD=\frac{C\times IR}{BW}$	(4)	*ADD* = average daily dose (mg/kg/day); *C =* heavy metals concentration detected in food products (mg/kg); *IR =* ingestion rate (g/day).	[174]
Average Daily Dose, ADDor Estimated Daily Intake, EDI	$ADD=\frac{C\times IR\times ED\times EF}{BW\times AT}$	(5)	*ADD* = average daily dose (mg/kg/day); *EDI* = estimated daily dose (mg/kg/day); *C* = heavy metals concentration detected in food products (mg/kg); *IR* = ingestion rate (g/day); *ED* = exposure duration (days/year); *EF* = exposure frequency (years); *BW* = body weight (kg); *AT* = averaging time (days).	[24,163,173,175]
	$EDI=\frac{C\times IR\times EF\times ED}{BW\times AT}$	(6)		
Hazard Quotient, HQ	$HQ=\frac{ADD}{RfD}$	(7)	*ADD* = average daily dose (mg/kg/day); *EDI* = estimated daily intake (mg/kg/day); *RfD* = reference dose (mg/kg/day).	[174]
	$HQ=\frac{EDI}{RfD}$	(8)		
Hazard Index, HI	$HI=\sum_{n=1}^{i}HQ$	(9)	*HQ* = Hazard Quotient.	[174]
Risk for carcinogenic chemical substances	Risk=LADD×SF	(10)	*LADD* = Lifetime Average Daily Dose, (mg/kg/day); *SF* = slope factor cancerogenic, [(mg/kg/day)^−1^].	[176]
Cumulative risk	$Cumulative\;risk=\overset{n}{\underset{i=1}{\sum }}Risk_{i}$	(11)	*Risk_i_* = Carcinogenic risk for chemical substance *i*.	[176]

**Table 4:** Hazard quotient (HQ) and hazard index (HI) of heavy metals in the medicinal plants or associated products.

Herbal/Herbal Mix/Product	Heavy Metals Detected		HQ	HI	Refs.
	Metal Ion	Concentration (mg/kg)			
*Aster tataricus* L.f.	Cr(III)	4.58	9.41 × 10^−5^	8.84 × 10^−2^	[175]
	Ni(II)	4.12	6.35 × 10^−3^		
	Cu(II)	24.73	1.91 × 10^−2^		
	Zn(II)	58.11	5.97 × 10^−3^		
	As(II)	0.19	1.91 × 10^−2^		
	Cd(II)	0.45	1.37 × 10^−2^		
	Hg(II)	0.20	8.72 × 10^−3^		
	Pb(II)	1.74	1.54 × 10^−2^		
*Salvia miltiorrhiza* Bge	Cr(III)	0.76	2.61 × 10^−5^	7.68 × 10^−2^	
	Ni(II)	4.53	1.16 × 10^−2^		
	Cu(II)	10.27	1.32 × 10^−2^		
	Zn(II)	14.92	2.55 × 10^−3^		
	As(II)	0.10	8.59 × 10^−3^		
	Cd(II)	0.06	3.02 × 10^−3^		
	Hg(II)	0.36	2.62 × 10^−2^		
	Pb(II)	0.79	1.16 × 10^−2^		
*Radix Aucklandiae*	Cr(III)	0.05	6.61 × 10^−3^	9.53 × 10^−2^	
	Ni(II)	5.27	4.87 × 10^−3^		
	Cu(II)	82.93	3.83 × 10^−2^		
	Zn(II)	50.49	3.11 × 10^−3^		
	As(II)	0.04	4.47 × 10^−3^		
	Cd(II)	0.24	4.47 × 10^−3^		
	Hg(II)	0.20	5.22 × 10^−3^		
	Pb(II)	7.01	3.71 × 10^−2^		
*Scutellaria baicalensis* Georgi	Cr(III)	0.28	5.06 × 10^−6^	3.87 × 10^−2^	
	Ni(II)	2.98	3.98 × 10^−3^		
	Cu(II)	13.56	9.06 × 10^−3^		
	Zn(II)	13.21	1.18 × 10^−3^		
	As(II)	0.03	3.04 × 10^−3^		
	Cd(II)	0.26	6.84 × 10^−3^		
	Hg(II)	0.22	8.07 × 10^−3^		
	Pb(II)	0.86	6.55 × 10^−3^		
*Aloe Percrassa, Verbascum sinaiticum*	Pb(II)	3.30	0.1269	0.7229	[177]
	Cr(III)	10.70	0.5487		
	Cu(II)	12.3	0.0473		
*Chenopodium murale*	Pb(II)	3.75	0.2875	1.228	
	Cr(III)	8.45	0.86		
	Cu(II)	10.5	0.0807		
*Urtica simensis, Trigonella Foenum-graceeum, Calpurnia aure*	Pb(II)	4.00	0.3075	1.472	
	Cr(III)	10.60	1.08		
	Cu(II)	11.05	0.0850		
*Verbena officinalis,* *Dodonaea angustifolia,* *Calpurnia aurea*	Pb(II)	4.00	0.3075	1.438	
	Cr(III)	10.15	1.04		
	Cu(II)	11.85	0.091		
*Carica papaya,* *Dodonaea angustifolia*	Pb(II)	3.00	0.23	0.776	
	Cr(III)	5.35	0.54		
	Cu(II)	0.81	0.006		
*Rumex abyssinicus,* *Trigonella Foenum-graceeum,* *Thymus vulgari*	Pb(II)	3.92	0.3	0.876	
	Cr(III)	5.60	0.57		
	Cu(II)	0.86	0.0065		
Argy Wormwood	Pb(II)	4.713	0.316	1.326	[178]
	Cd(II)	1.051	0.247		
	As(II)	0.884	0.692		
	Hg(II)	0.027	0.063		
	Cu(II)	16.39	0.008		
Plantain Herb	Pb(II)	3.110	0.209	1.541	
	Cd(II)	0.269	0.063		
	As(II)	1.506	1.179		
	Hg(II)	0.036	0.085		
	Cu(II)	11.44	0.005		
Peppermint	Pb(II)	1.836	0.123	0.653	
	Cd(II)	0.116	0.027		
	As(II)	0.329	0.257		
	Hg(II)	0.103	0.242		
	Cu(II)	9.254	0.004		
Rhubarb	Pb(II)	0.340	0.023	0.159	
	Cd(II)	0.086	0.020		
	As(II)	0.112	0.088		
	Hg(II)	0.011	0.026		
	Cu(II)	3.551	0.002		
Chrysanthemum Flower	Pb(II)	0.888	0.060	1.146	
	Cd(II)	0.257	0.060		
	As(II)	0.270	0.211		
	Hg(II)	0.345	0.810		
	Cu(II)	9.903	0.005		
Common Coltsfoot Flower	Pb(II)	1.173	0.079	0.651	
	Cd(II)	0.081	0.019		
	As(II)	0.647	0.506		
	Hg(II)	0.018	0.042		
	Cu(II)	10.30	0.005		
Turmeric Root Tuber	Pb(II)	0.580	0.039	0.159	
	Cd(II)	0.179	0.042		
	As(II)	0.087	0.068		
	Hg(II)	0.004	0.009		
	Cu(II)	2.348	0.001		
*Hibiscus sabdariffa*	Zn(II)	4.10	0.77	2.07	[179]
	Pb(II)	0.74	0.13		
	Cd(II)	0.10	0.10		
	Ni(II)	0.39	0.29		
	Cu(II)	0.08	0.01		
	Fe(II)	7.9	0.77		
*Curcuma longa*	Zn(II)	5.20	1.00	2.52	
	Pb(II)	0.79	0.15		
	Cd(II)	0.12	0.12		
	Ni(II)	0.37	0.25		
	Cu(II)	0.26	0.04		
	Fe(II)	9.8	0.96		
*Ocimum basilicum*	Zn(II)	5.18	0.99	2.30	
	Pb(II)	0.74	0.13		
	Cd(II)	0.16	0.16		
	Ni(II)	0.38	0.27		
	Cu(II)	0.12	0.02		
	Fe(II)	7.5	0.73		
*Allium sativum*	Zn(II)	5.60	1.04	2.44	
	Pb(II)	0.62	0.10		
	Cd(II)	0.13	0.13		
	Ni(II)	0.37	0.25		
	Cu(II)	0.07	0.01		
	Fe(II)	9.3	0.91		
*Zingiber officinale*	Zn(II)	7.20	1.39	2.69	
	Pb(II)	2.75	0.51		
	Cd(II)	0.17	0.17		
	Ni(II)	0.63	0.48		
	Cu(II)	0.51	0.11		
	Fe(II)	0.3	0.03		
*Asprellae ilicis radix*	Pb(II)	0.0126	1.76	2.65	[180]
	Cd(II)	0.00178	0.89		
Hedyotidis diffusae herba	Pb(II)	0.00302	0.85	2.72	
	As(II)	0.00088	0.41		
	Cd(II)	0.00146	1.46		
Plantaginis herba	Pb(II)	0.00264	0.37	1.00	
	As(II)	0.00239	0.56		
	Cd(II)	0.00014	0.07		
Lysimachiae herba	Pb(II)	0.00323	0.9	1.53	
	As(II)	0.00065	0.3		
	Cd(II)	0.00032	0.32		
Violae herba	Pb(II)	0.00464	0.65	1.24	
	As(II)	0.00175	0.41		
	Cd(II)	0.00036	0.18		
Centipedae herba	Pb(II)	0.00673	0.38	1.00	
	As(II)	0.00168	0.16		
	Cd(II)	0.00232	0.46		
Eckloniae/Laminariae thallus	Pb(II)	0.00267	0.15	3.42	
	As(II)	0.0337	3.13		
	Cd(II)	0.0007	0.14		
Toxicodendri resina	Pb(II)	0.0738	1.55	11.9	
	As(II)	0.00478	0.17		
	Cd(II)	0.00055	0.04		
	Hg(II)	0.0977	10.2		
Pheretima	Pb(II)	0.0141	0.66	1.15	
	As(II)	0.0024	0.19		
	Cd(II)	0.00164	0.27		
	Hg(II)	0.00015	0.03		
Fossilia Ossis Mastodi	Pb(II)	0.0054	0.76	2.19	
	As(II)	0.0056	1.3		
	Cd(II)	0.00026	0.13		
Haematitum	Pb(II)	0.00559	0.78	2.08	
	As(II)	0.00525	1.22		
	Hg(II)	0.00011	0.08		
Tea bags	Fe(II)	1.05–7.45	2.00 × 10^−4^–1.42 × 10^−3^	0.68–1.11	[181]
	Zn(II)	0.10–0.30	4.44 × 10^−5^–1.33 × 10^−3^		
	As(II)	1.40–2.00	0.62–0.89		
	Cd(II)	0.10–1.50	0.01–0.20		
	Pb(II)	0.10–0.40	3.81 × 10^−3^–1.52 × 10^−3^		

## Data Availability

Not applicable.
